# Supplementary material for: Ageing Investigation Using Two-Time-Point Metabolomics Data from KORA and CARLA Studies
Source: Metabolites. 2019 Mar 5;9(3):44. doi: 10.3390/metabo9030044 (PMC6468431; doi:10.3390/metabo9030044)
Supplement: Supplementary file 1 [file metabolites-09-00044-s001.pdf]

Ageing investigation using two-time-point metabolomics data from KORA and CARLA studies

Supplementary Materials:

Table S1. The list of 123 metabolites measured and passed quality control in KORA S4 and F4 and in comparison with the median values from literature.

The list shows all 123 metabolites measured in both KORA S4 and F4 and in comparison with the median values from literature. The coefficient of variation (CV) of each metabolite measured from the reference samples of Absolute*IDQ*™ p180 Kit and Absolute*IDQ*™ p150 Kit are shown respectively. A total of 122 metabolites passed quality control (CV < 25% in both time-points). The medians (in μM) of 122 metabolites measured with Absolute*IDQ*™ p180 Kit (used in KORA S4) and Absolute*IDQ*™ p150 Kit (used in KORA F4) using the same reference human plasma samples are shown below. To ensure normalization quality, outliers with metabolite concentration of over the range of median ± 1.5 interquartile range were excluded before median values of the reference samples were calculated. The normalization factor (NF) for each metabolite equals to the median values of the reference samples measured in Absolute*IDQ*™ p180 Kit divided by the median values measured in Absolute*IDQ*™ p150 Kit. Then, the metabolite concentration of each sample in KORA F4 was adjusted by multiplying the concentration values to the NF of their corresponding metabolites. CV, coefficient of variation. ND, not detected. NF, normalization factor.

Metabolite	Biochemical name	CV	CV	Median	Median	Median	Median	NF	Applicati
C0	Carnitine	5.71	7.48	34.50	36.10	35.2	-	0.96	Used
C2	Acetylcarnitine	6.36	6.64	4.15	4.17	6.13	6.81	1.00	Used
C3	Propionylcarnitine	9.95	9.70	0.36	0.38	0.32	0.45	0.96	Used
C4	Butyrylcarnitine	9.54	10.59	0.16	0.17	0.19	0.25	0.94	Used
C6 (C4:1-DC)	Hexanoylcarnitine (Fumarylcarnitine)	10.85	14.12	0.14	0.13	0.06	-	1.07	Used
C5	Valerylcarnitine	20.39	18.98	0.05	0.04	0.14	0.18	1.09	Used
C8	Octanoylcarnitine	12.91	14.37	0.13	0.13	0.16	0.14	1.06	Used
C9	Nonaylcarnitine	22.84	36.96	0.02	0.03	0.07	-	-	Excluded
C10	Decanoylcarnitine	11.33	15.20	0.16	0.16	0.28	0.17	0.96	Used
C10:1	Decenoylcarnitine	10.77	13.43	0.12	0.14	ND	0.15	0.85	Used
C10:2	Decadienylcarnitine	16.13	16.43	0.03	0.04	ND	0.04	0.90	Used
C12	Dodecanoylcarnitine	11.02	14.85	0.07	0.06	ND	0.08	1.15	Used
C14	Tetradecanoylcarnitine	15.84	20.39	0.03	0.03	ND	0.03	1.01	Used
C14:1	Tetradecenoylcarnitine	11.44	19.36	0.07	0.06	0.12	0.07	1.03	Used
C14:2	Tetradecadienylcarnitine	18.02	19.40	0.02	0.02	0.03	33.8	0.99	Used
C16	Hexadecanoylcarnitine	10.99	12.26	0.07	0.07	0.10	0.07	1.01	Used
C16:1	Hexadecenoylcarnitine	18.06	19.42	0.02	0.03	ND	0.02	0.92	Used
C18	Octadecanoylcarnitine	15.44	23.26	0.03	0.03	0.04	0.04	1.00	Used
C18:1	Octadecenoylcarnitine	9.60	12.10	0.08	0.09	0.11	0.12	0.91	Used
C18:2	Octadecadienylcarnitine	10.44	12.83	0.05	0.05	0.05	0.06	0.97	Used
PC aa C28:1	Phosphatidylcholine diacyl C28:1	9.34	8.18	1.52	1.51	2.91	-	1.01	Used
PC aa C30:0	Phosphatidylcholine diacyl C30:0	9.27	8.01	1.96	1.90	4.44	-	1.03	Used
PC aa C32:0	Phosphatidylcholine diacyl C32:0	8.19	7.59	6.75	6.83	14.8	-	0.99	Used
PC aa C32:1	Phosphatidylcholine diacyl C32:1	9.11	9.06	4.33	4.31	14.4	-	1.01	Used
PC aa C32:2	Phosphatidylcholine diacyl C32:2	11.78	20.84	1.33	1.23	3.84	-	1.08	Used
PC aa C32:3	Phosphatidylcholine diacyl C32:3	9.02	9.36	0.42	0.40	0.47	-	1.05	Used
PC aa C34:1	Phosphatidylcholine diacyl C34:1	6.80	6.85	89.00	90.35	234.5	-	0.99	Used
PC aa C34:2	Phosphatidylcholine diacyl C34:2	6.76	8.26	224.00	239.00	407.6	-	0.94	Used
PC aa C34:3	Phosphatidylcholine diacyl C34:3	6.09	6.94	8.73	8.91	15.1	-	0.98	Used
PC aa C34:4	Phosphatidylcholine diacyl C34:4	6.74	8.86	0.93	0.94	1.77	-	0.99	Used
PC aa C36:0	Phosphatidylcholine diacyl C36:0	11.09	11.14	1.68	1.93	2.42	-	0.87	Used
PC aa C36:1	Phosphatidylcholine diacyl C36:1	6.80	8.23	24.00	24.90	55.3	-	0.96	Used
PC aa C36:2	Phosphatidylcholine diacyl C36:2	6.36	7.32	153.00	165.00	245.5	-	0.93	Used
PC aa C36:3	Phosphatidylcholine diacyl C36:3	6.27	6.60	82.60	88.25	127.2	-	0.94	Used
PC aa C36:4	Phosphatidylcholine diacyl C36:4	6.13	6.68	136.00	143.00	184.3	-	0.95	Used
PC aa C36:5	Phosphatidylcholine diacyl C36:5	6.49	6.68	10.60	11.10	25.8	-	0.95	Used
PC aa C36:6	Phosphatidylcholine diacyl C36:6	8.98	11.20	0.45	0.47	1.10	-	0.95	Used
PC aa C38:0	Phosphatidylcholine diacyl C38:0	8.66	7.92	1.81	1.93	3.36	-	0.94	Used
PC aa C38:3	Phosphatidylcholine diacyl C38:3	6.75	6.50	33.20	35.40	45.2	-	0.94	Used
PC aa C38:4	Phosphatidylcholine diacyl C38:4	5.56	6.54	88.60	95.65	99.1	-	0.93	Used
PC aa C38:5	Phosphatidylcholine diacyl C38:5	5.56	6.54	39.00	41.10	52.2	-	0.95	Used
PC aa C38:6	Phosphatidylcholine diacyl C38:6	6.76	6.66	38.90	40.90	86.5	-	0.95	Used
PC aa C40:2	Phosphatidylcholine diacyl C40:2	14.48	12.64	0.37	0.39	0.29	-	0.93	Used
PC aa C40:3	Phosphatidylcholine diacyl C40:3	13.79	12.56	0.54	0.56	0.52	-	0.96	Used
PC aa C40:4	Phosphatidylcholine diacyl C40:4	6.69	7.62	3.48	3.76	3.05	-	0.93	Used
PC aa C40:5	Phosphatidylcholine diacyl C40:5	6.32	6.51	8.59	9.22	8.99	-	0.93	Used
PC aa C40:6	Phosphatidylcholine diacyl C40:6	5.96	6.62	15.90	16.20	26.5	-	0.98	Used

PC aa C42:0	Phosphatidylcholine diacyl C42:0	9.22	12.66	0.43	0.42	0.63	-	1.02	Used
PC aa C42:1	Phosphatidylcholine diacyl C42:1	11.92	11.29	0.21	0.22	0.31	-	0.96	Used
PC aa C42:2	Phosphatidylcholine diacyl C42:2	13.42	13.14	0.17	0.18	0.24	-	0.98	Used
PC aa C42:4	Phosphatidylcholine diacyl C42:4	11.12	11.99	0.23	0.23	0.18	-	0.99	Used
PC aa C42:5	Phosphatidylcholine diacyl C42:5	11.12	11.38	0.30	0.31	0.30	-	0.96	Used
PC aa C42:6	Phosphatidylcholine diacyl C42:6	11.83	11.30	0.41	0.47	0.45	-	0.87	Used
PC ae C30:0	Phosphatidylcholine acyl-alkyl C30:0	18.10	16.02	0.17	0.18	0.49	-	0.93	Used
PC ae C32:1	Phosphatidylcholine acyl-alkyl C32:1	8.97	8.83	1.43	1.40	2.99	-	1.02	Used
PC ae C32:2	Phosphatidylcholine acyl-alkyl C32:2	12.17	9.88	0.37	0.37	0.76	-	1.01	Used
PC ae C34:0	Phosphatidylcholine acyl-alkyl C34:0	9.42	9.46	0.64	0.65	1.62	-	0.98	Used
PC ae C34:1	Phosphatidylcholine acyl-alkyl C34:1	7.20	7.34	4.07	4.07	11.2	-	1.00	Used
PC ae C34:2	Phosphatidylcholine acyl-alkyl C34:2	6.97	7.04	6.04	6.07	12.9	-	1.00	Used
PC ae C34:3	Phosphatidylcholine acyl-alkyl C34:3	6.76	6.75	5.30	5.23	9.03	-	1.01	Used
PC ae C36:1	Phosphatidylcholine acyl-alkyl C36:1	7.33	7.88	5.46	5.94	9.47	-	0.92	Used
PC ae C36:2	Phosphatidylcholine acyl-alkyl C36:2	6.84	6.83	7.26	7.52	16.5	-	0.97	Used
PC ae C36:3	Phosphatidylcholine acyl-alkyl C36:3	6.88	7.73	4.50	4.59	8.32	-	0.98	Used
PC ae C36:4	Phosphatidylcholine acyl-alkyl C36:4	5.96	6.65	12.00	12.40	20.0	-	0.97	Used
PC ae C36:5	Phosphatidylcholine acyl-alkyl C36:5	5.80	6.59	9.37	9.64	14.2	-	0.97	Used
PC ae C38:0	Phosphatidylcholine acyl-alkyl C38:0	8.06	8.92	1.41	1.62	2.46	-	0.87	Used
PC ae C38:1	Phosphatidylcholine acyl-alkyl C38:1	14.68	14.06	1.49	1.68	0.73	-	0.89	Used
PC ae C38:2	Phosphatidylcholine acyl-alkyl C38:2	11.66	9.61	2.23	2.42	1.95	-	0.92	Used
PC ae C38:3	Phosphatidylcholine acyl-alkyl C38:3	6.90	8.00	5.42	6.01	4.33	-	0.90	Used
PC ae C38:4	Phosphatidylcholine acyl-alkyl C38:4	5.94	6.62	9.86	10.80	14.5	-	0.91	Used
PC ae C38:5	Phosphatidylcholine acyl-alkyl C38:5	5.78	6.45	12.60	13.40	19.0	-	0.94	Used
PC ae C38:6	Phosphatidylcholine acyl-alkyl C38:6	6.21	6.56	5.11	5.38	8.73	-	0.95	Used
PC ae C40:1	Phosphatidylcholine acyl-alkyl C40:1	10.93	11.32	1.04	1.06	1.41	-	0.98	Used
PC ae C40:2	Phosphatidylcholine acyl-alkyl C40:2	8.02	7.94	1.47	1.61	2.12	-	0.92	Used
PC ae C40:3	Phosphatidylcholine acyl-alkyl C40:3	8.81	7.40	3.35	3.68	1.12	-	0.91	Used
PC ae C40:4	Phosphatidylcholine acyl-alkyl C40:4	8.39	8.19	3.24	3.38	2.35	-	0.96	Used
PC ae C40:5	Phosphatidylcholine acyl-alkyl C40:5	6.27	7.46	4.24	4.82	3.67	-	0.88	Used
PC ae C40:6	Phosphatidylcholine acyl-alkyl C40:6	6.65	7.14	2.98	3.27	5.23	-	0.91	Used
PC ae C42:1	Phosphatidylcholine acyl-alkyl C42:1	16.00	13.06	0.40	0.41	0.33	-	0.96	Used
PC ae C42:2	Phosphatidylcholine acyl-alkyl C42:2	11.38	11.01	0.41	0.44	0.62	-	0.94	Used
PC ae C42:3	Phosphatidylcholine acyl-alkyl C42:3	9.63	9.09	0.65	0.67	0.81	-	0.97	Used
PC ae C42:4	Phosphatidylcholine acyl-alkyl C42:4	7.66	8.30	1.00	1.04	0.90	-	0.96	Used
PC ae C42:5	Phosphatidylcholine acyl-alkyl C42:5	7.32	6.72	2.27	2.50	2.16	-	0.91	Used
PC ae C44:3	Phosphatidylcholine acyl-alkyl C44:3	23.41	17.24	0.12	0.12	0.11	-	0.97	Used
PC ae C44:4	Phosphatidylcholine acyl-alkyl C44:4	12.21	13.79	0.26	0.28	0.38	-	0.93	Used
PC ae C44:5	Phosphatidylcholine acyl-alkyl C44:5	7.35	7.85	1.03	1.06	1.88	-	0.97	Used
PC ae C44:6	Phosphatidylcholine acyl-alkyl C44:6	7.55	7.93	1.06	1.05	1.29	-	1.01	Used
LysoPC a	Lysophosphatidylcholine acyl C16:0	6.73	12.50	191.00	188.00	71.7	-	1.02	Used
LysoPC a	Lysophosphatidylcholine acyl C16:1	6.92	10.60	3.78	3.77	2.04	-	1.00	Used
LysoPC a	Lysophosphatidylcholine acyl C17:0	7.27	11.24	3.09	3.26	1.60	-	0.95	Used
LysoPC a	Lysophosphatidylcholine acyl C18:0	7.07	11.35	64.30	66.65	21.4	-	0.96	Used
LysoPC a	Lysophosphatidylcholine acyl C18:1	6.70	10.53	26.70	27.25	16.9	-	0.98	Used
LysoPC a	Lysophosphatidylcholine acyl C18:2	6.92	13.19	28.20	29.00	25.3	-	0.97	Used
LysoPC a	Lysophosphatidylcholine acyl C20:3	8.62	13.65	2.58	2.84	1.65	-	0.91	Used
LysoPC a	Lysophosphatidylcholine acyl C20:4	7.33	12.93	10.80	11.80	4.99	-	0.92	Used
SM (OH) C14:1	Hydroxysphingomyeline C14:1	10.68	11.03	4.92	4.10	6.90	-	1.20	Used
SM (OH) C16:1	Hydroxysphingomyeline C16:1	10.62	11.74	3.13	2.52	3.99	-	1.24	Used
SM (OH) C22:1	Hydroxysphingomyeline C22:1	10.89	11.84	13.90	11.90	15.1	-	1.17	Used
SM (OH) C22:2	Hydroxysphingomyeline C22:2	10.71	12.52	8.67	7.38	13.6	-	1.17	Used
SM (OH) C24:1	Hydroxysphingomyeline C24:1	14.54	13.10	1.30	1.24	1.51	-	1.05	Used
SM C16:0	Sphingomyeline C16:0	10.42	10.91	102.00	83.35	113.9	-	1.22	Used
SM C16:1	Sphingomyeline C16:1	9.52	10.49	15.50	12.90	16.3	-	1.20	Used
SM C18:0	Sphingomyeline C18:0	9.59	11.03	22.80	18.70	23.4	-	1.22	Used
SM C18:1	Sphingomyeline C18:1	9.09	10.29	12.30	10.20	10.7	-	1.21	Used
SM C20:2	Sphingomyeline C20:2	15.99	15.52	0.57	0.54	0.34	-	1.05	Used
SM C24:0	Sphingomyeline C24:0	11.54	11.90	22.30	20.35	21.7	-	1.10	Used
SM C24:1	Sphingomyeline C24:1	11.84	12.39	48.40	41.90	56.2	-	1.16	Used
SM C26:1	Sphingomyeline C26:1	20.92	20.50	0.34	0.31	0.46	-	1.09	Used
H1	Sum of Hexoses	5.18	5.54	4791.00	5051.50	5088	-	0.95	Used
Arg	Arginine	12.58	8.23	94.10	106.00	80.7	125.8	0.89	Used
Gln	Glutamine	12.46	8.56	255.00	279.00	652.9	100.0	0.91	Used
Gly	Glycine	12.41	8.39	207.00	239.00	244.6	313.2	0.87	Used
His	Histidine	12.52	10.80	69.70	67.10	89.4	86.7	1.04	Used
Met	Methionine	12.82	12.34	25.90	38.70	24.9	31.4	0.67	Used
Orn	Ornithine	14.51	9.73	60.30	68.00	66.1	77.5	0.89	Used
Phe	Phenylalanine	11.59	8.48	53.30	49.65	59.4	76.2	1.07	Used
Pro	Proline	11.09	7.66	215.00	235.00	179.4	231.6	0.91	Used
Ser	Serine	13.47	15.19	89.90	86.25	112.6	116.6	1.04	Used
Thr	Threonine	18.33	17.35	145.00	112.00	124.5	-	1.29	Used
Trp	Tryptophan	12.31	8.89	43.70	64.60	62.7	-	0.68	Used
Tyr	Tyrosine	13.87	9.06	59.80	64.80	61.4	82.7	0.92	Used
Val	Valine	13.00	10.07	179.00	233.50	241.1	275.4	0.77	Used
xLeu	Sum of Leucine and Isoleucine	12.57	9.45	129.10	191.00	224.1	196.1	0.68	Used

Table S2. Changes in metabolite concentration in KORA Study in women (*N* = 317) and men (*N* = 273) over seven-year period.

Beta estimates (ß) and confidence interval (CI) of changes in metabolite concentration every year were calculated using multivariate generalised estimation equation models. The model was adjusted for chronological age at baseline, body mass index, physical activity, smoking status, alcohol intake and systolic blood pressure. Significant P values after Bonferroni correction (cut-offs: P value $< \frac{0.05}{122} = 4.1 \times 10^{-4}$) and their false discovery rate-adjusted P values (pFDR) at 5% level were highlighted in bold. xLeucine, sum of leucine and isoleucine.

	**Women in KORA (n = 317)**			**Men in KORA (n = 273)**		
**Metabolites**	ß (95% CI)	P value	pFDR	ß (95% CI)	P value	pFDR
**C0**	-0.07 (-0.08, -0.05)	**2.09×10^{-20}**	**7.98×10^{-20}**	-0.1 (-0.12, 0.09)	**8.03×10^{-40}**	**5.44×10^{-39}**
**C2**	0.06 (0.04, 0.07)	**1.71×10^{-11}**	**4.26×10^{-11}**	0.04 (0.02, 0.06)	**4.56×10^{-6}**	**8.06×10^{-6}**
**C3**	-0.06 (-0.07, -0.04)	**9.14×10^{-15}**	**3.09×10^{-14}**	-0.08 (-0.1, -0.07)	**6.98×10^{-26}**	**2.94×10^{-25}**
**C4**	0.03 (0.01, 0.04)	**3.02×10^{-5}**	**5.58×10^{-5}**	0 (-0.01, 0.01)	0.94	0.94
C6 (C4:1-DC)	0.01 (0, 0.03)	0.11	0.11	0 (-0.02, 0.01)	0.79	0.82
**C5**	-0.09 (-0.11, -0.08)	**6.00×10^{-24}**	**2.61×10^{-23}**	-0.1 (-0.11, 0.08)	**6.62×10^{-34}**	**3.67×10^{-33}**
C8	0.02 (0, 0.03)	0.06	0.07	0 (-0.01, 0.02)	0.69	0.72
C10	0.03 (0.01, 0.04)	3.05×10^{-3}	**4.59×10^{-3}**	0.01 (-0.01, 0.02)	0.50	0.55
**C10:1**	-0.02 (-0.03, 0)	0.07	0.09	-0.04 (-0.05, 0.02)	**5.44×10^{-5}**	**8.85×10^{-5}**
C10:2	-0.01 (-0.02, 0.01)	0.54	0.59	-0.03 (-0.05, 0)	0.02	0.03
**C12**	0.06 (0.04, 0.07)	**9.30×10^{-10}**	**2.03×10^{-9}**	0.03 (0.02, 0.05)	**7.26×10^{-5}**	**1.17×10^{-4}**
**C14**	-0.03 (-0.05, -0.01)	7.11×10^{-4}	**1.17×10^{-3}**	-0.05 (-0.07, 0.03)	**2.82×10^{-7}**	**5.74×10^{-7}**
**C14:1**	0.09 (0.07, 0.11)	**1.35×10^{-22}**	**5.68×10^{-22}**	0.07 (0.05, 0.09)	**1.34×10^{-12}**	**3.62×10^{-12}**
**C14:2**	-0.01 (-0.03, 0.01)	0.44	0.49	-0.03 (-0.05, 0.02)	**1.05×10^{-4}**	**1.67×10^{-4}**
C16	-0.01 (-0.03, 0.01)	0.26	0.29	-0.02 (-0.04, 0)	0.02	**0.02**
**C16:1**	-0.03 (-0.05, -0.01)	4.69×10^{-4}	**7.84×10^{-4}**	-0.05 (-0.06, 0.03)	**3.78×10^{-6}**	**6.88×10^{-6}**
**C18**	0.03 (0.01, 0.05)	**3.68×10^{-4}**	**6.24×10^{-4}**	0.01 (-0.01, 0.03)	2.87×10^{-1}	3.33×10^{-1}
**C18:1**	-0.03 (-0.04, -0.01)	3.73×10^{-3}	**5.49×10^{-3}**	-0.04 (-0.06, 0.03)	**1.74×10^{-6}**	**3.37×10^{-6}**
C18:2	0 (-0.01, 0.02)	0.71	0.74	-0.01 (-0.03, 0.01)	0.23	0.28
PC aa C28:1	0 (-0.01, 0.02)	0.65	0.69	-0.01 (-0.03, 0)	0.10	0.13
**PC aa C30:0**	-0.07 (-0.08, -0.05)	**2.70×10^{-13}**	**8.04×10^{-13}**	-0.07 (-0.09, 0.06)	**9.28×10^{-18}**	**3.06×10^{-17}**
**PC aa C32:0**	0.05 (0.03, 0.06)	**7.85×10^{-11}**	**1.88×10^{-10}**	0.04 (0.03, 0.06)	**7.01×10^{-7}**	**1.38×10^{-6}**
**PC aa C32:1**	0.04 (0.03, 0.06)	**7.62×10^{-10}**	**1.69×10^{-09}**	0.04 (0.03, 0.06)	**1.03×10^{-7}**	**2.17×10^{-7}**
PC aa C32:2	-0.01 (-0.03, 0.01)	0.24	0.27	-0.01 (-0.02, 0.01)	0.48	0.53
PC aa C32:3	-0.02 (-0.04, -0.01)	2.12×10^{-3}	**3.28×10^{-3}**	-0.02 (-0.04, 0.01)	4.64×10^{-3}	**6.74×10^{-3}**
**PC aa C34:1**	0.05 (0.03, 0.07)	**3.44×10^{-10}**	**7.76×10^{-10}**	0.05 (0.04, 0.07)	**9.48×10^{-10}**	**2.31×10^{-9}**
PC aa C34:2	-0.01 (-0.03, 0.01)	0.19	0.22	-0.02 (-0.03, 0)	0.13	0.16
PC aa C34:3	0 (-0.02, 0.01)	0.68	0.71	-0.01 (-0.02, 0.01)	0.46	0.51
PC aa C34:4	0.02 (0, 0.04)	0.02	0.03	0.01 (-0.01, 0.02)	0.31	0.35
**PC aa C36:0**	-0.11 (-0.12, -0.09)	**8.09×10^{-50}**	**7.05×10^{-49}**	-0.12 (-0.13, 0.1)	**2.13×10^{-44}**	**1.73×10^{-43}**
PC aa C36:1	0.01 (0, 0.03)	8.48×10^{-2}	1.08×10^{-1}	0 (-0.01, 0.02)	8.66×10^{-1}	8.88×10^{-1}
**PC aa C36:2**	-0.1 (-0.12, -0.09)	**9.07×10^{-43}**	**5.82×10^{-42}**	-0.13 (-0.14, -0.11)	**1.89×10^{-52}**	**2.10×10^{-51}**
**PC aa C36:3**	-0.05 (-0.06, -0.03)	**5.74×10^{-9}**	**1.19×10^{-8}**	-0.05 (-0.07, -0.03)	**2.15×10^{-8}**	**4.86×10^{-8}**
PC aa C36:4	0 (-0.01, 0.02)	0.65	0.69	0 (-0.02, 0.01)	0.68	0.72
PC aa C36:5	-0.01 (-0.03, 0)	0.17	0.20	-0.02 (-0.04, 0)	0.02	0.03
PC aa C36:6	-0.01 (-0.03, 0)	0.09	0.11	-0.02 (-0.04, 0)	0.01	0.02
**PC aa C38:0**	-0.03 (-0.05, -0.02)	**1.47×10^{-6}**	**2.90×10^{-6}**	-0.04 (-0.06, -0.03)	**1.44×10^{-7}**	**2.97×10^{-7}**
**PC aa C38:3**	-0.03 (-0.05, -0.02)	**1.35×10^{-5}**	**2.57×10^{-5}**	-0.05 (-0.07, -0.04)	**5.33×10^{-11}**	**1.36×10^{-10}**
**PC aa C38:4**	0 (-0.02, 0.01)	0.48	0.53	-0.03 (-0.04, -0.01)	**2.81×10^{-4}**	**4.34×10^{-4}**
PC aa C38:5	-0.01 (-0.02, 0)	0.20	0.24	-0.03 (-0.04, -0.01)	6.90×10^{-4}	**1.03×10^{-3}**
PC aa C38:6	-0.01 (-0.03, 0)	0.09	0.11	-0.02 (-0.03, 0)	0.06	0.08
PC aa C40:2	-0.01 (-0.03, 0)	0.12	0.15	-0.01 (-0.03, 0.02)	0.56	0.61
PC aa C40:3	-0.01 (-0.03, 0)	0.16	0.20	0 (-0.02, 0.02)	0.94	0.94
PC aa C40:4	0 (-0.02, 0.01)	0.60	0.65	-0.02 (-0.04, 0)	0.03	0.05
**PC aa C40:5**	-0.05 (-0.06, -0.03)	**3.78×10^{-9}**	**7.96×10^{-9}**	-0.08 (-0.09, -0.06)	**5.08×10^{-24}**	**2.07×10^{-23}**
**PC aa C40:6**	-0.03 (-0.04, -0.01)	**1.21×10^{-4}**	**2.13×10^{-4}**	-0.05 (-0.06, -0.03)	**7.57×10^{-10}**	**1.89×10^{-9}**
**PC aa C42:0**	0.05 (0.04, 0.06)	**9.36×10^{-15}**	**3.09×10^{-14}**	0.05 (0.04, 0.07)	**5.05×10^{-12}**	**1.31×10^{-11}**
**PC aa C42:1**	0.04 (0.03, 0.06)	**2.36×10^{-8}**	**4.79×10^{-8}**	0.04 (0.02, 0.05)	**1.78×10^{-6}**	**3.39×10^{-6}**
PC aa C42:2	0.02 (0, 0.04)	0.02	**0.02**	0.02 (0, 0.04)	0.06	0.08
**PC aa C42:4**	0.06 (0.05, 0.08)	**1.19×10^{-12}**	**3.22×10^{-12}**	0.08 (0.06, 0.1)	**1.13×10^{-14}**	**3.21×10^{-14}**

PC aa C42:5	0 (-0.02, 0.01)	0.61	0.65	0.01 (-0.01, 0.03)	0.28	0.33
**PC aa C42:6**	-0.05 (-0.07, -0.04)	**9.98×10^{-11}**	**2.34×10^{-10}**	-0.04 (-0.06, -0.02)	**3.98×10^{-6}**	**7.15×10^{-6}**
PC ae C30:0	-0.03 (-0.04, -0.01)	3.51×10^{-3}	**5.22×10^{-3}**	0 (-0.02, 0.02)	0.93	0.94
**PC ae C32:1**	0.04 (0.03, 0.06)	**1.14×10^{-10}**	**2.62×10^{-10}**	0.06 (0.04, 0.07)	**2.30×10^{-12}**	**6.09×10^{-12}**
**PC ae C32:2**	0.06 (0.05, 0.07)	**3.76×10^{-21}**	**1.48×10^{-20}**	0.07 (0.06, 0.09)	**6.45×10^{-20}**	**2.32×10^{-19}**
**PC ae C34:0**	0.04 (0.02, 0.05)	**5.57×10^{-6}**	**1.08×10^{-5}**	0.04 (0.02, 0.05)	**3.00×10^{-5}**	**5.08×10^{-5}**
**PC ae C34:1**	0.02 (0.01, 0.04)	2.57×10^{-3}	**3.91×10^{-3}**	0.04 (0.02, 0.05)	**7.58×10^{-6}**	**1.32×10^{-5}**
**PC ae C34:2**	0.02 (0, 0.03)	0.02	**0.03**	0.04 (0.02, 0.06)	**3.11×10^{-6}**	**5.74×10^{-6}**
**PC ae C34:3**	0.05 (0.03, 0.06)	**5.90×10^{-12}**	**1.50×10^{-11}**	0.06 (0.04, 0.07)	**2.05×10^{-14}**	**5.69×10^{-14}**
**PC ae C36:1**	-0.05 (-0.07, -0.04)	**1.91×10^{-12}**	**5.08×10^{-12}**	-0.05 (-0.06, -0.03)	**3.88×10^{-9}**	**9.29×10^{-9}**
PC ae C36:2	-0.01 (-0.02, 0)	0.17	0.20	0 (-0.02, 0.01)	0.67	0.72
**PC ae C36:3**	0.03 (0.01, 0.04)	**2.50×10^{-4}**	**4.36×10^{-4}**	0.05 (0.03, 0.06)	**5.51×10^{-8}**	**1.20×10^{-7}**
PC ae C36:4	0 (-0.01, 0.01)	0.92	0.92	0.01 (-0.01, 0.03)	0.35	0.40
**PC ae C36:5**	0.04 (0.03, 0.06)	**4.39×10^{-11}**	**1.07×10^{-10}**	0.03 (0.01, 0.05)	**3.09×10^{-4}**	**4.71×10^{-4}**
PC ae C38:0	-0.02 (-0.0, 0)	0.01	0.02	-0.02 (-0.04, -0.01)	6.07×10^{-3}	**8.51×10^{-3}**
**PC ae C38:1**	0.08 (0.06, 0.1)	**1.69×10^{-14}**	**5.29×10^{-14}**	0.09 (0.07, 0.11)	**5.85×10^{-17}**	**1.83×10^{-16}**
PC ae C38:2	-0.02 (-0.03, 0)	0.04	0.05	-0.01 (-0.03, 0.01)	0.20	0.25
**PC ae C38:3**	-0.03 (-0.05, -0.02)	**6.45×10^{-5}**	**1.16×10^{-4}**	-0.03 (-0.04, -0.01)	1.09×10^{-3}	**1.60×10^{-3}**
**PC ae C38:4**	-0.04 (-0.06, -0.03)	**2.93×10^{-9}**	**6.26×10^{-9}**	-0.04 (-0.05, -0.02)	**3.63×10^{-5}**	**6.07×10^{-5}**
PC ae C38:5	0 (-0.02, 0.01)	0.80	0.82	-0.01 (-0.03, 0.01)	0.25	0.30
**PC ae C38:6**	-0.02 (-0.03, 0)	0.01	**0.02**	-0.03 (-0.05, -0.01)	**3.74×10^{-4}**	**5.63×10^{-4}**
PC ae C40:1	0.02 (0.01, 0.04)	3.85×10^{-3}	**5.59×10^{-3}**	0.01 (-0.01, 0.03)	1.60×10^{-1}	1.99×10^{-1}
**PC ae C40:2**	-0.02 (-0.04, -0.01)	1.07×10^{-3}	**1.72×10^{-3}**	-0.03 (-0.05, -0.02)	**1.53×10^{-4}**	**2.39×10^{-4}**
**PC ae C40:3**	-0.07 (-0.08, -0.05)	**5.17×10^{-18}**	**1.91×10^{-17}**	-0.05 (-0.07, -0.03)	**5.70×10^{-9}**	**1.34×10^{-8}**
**PC ae C40:4**	-0.05 (-0.07, -0.04)	**4.13×10^{-13}**	**1.20×10^{-12}**	-0.04 (-0.06, -0.02)	**2.68×10^{-5}**	**4.61×10^{-5}**
**PC ae C40:5**	-0.09 (-0.11, -0.08)	**5.07×10^{-40}**	**3.09×10^{-39}**	-0.09 (-0.11, -0.08)	**1.16×10^{-31}**	**5.65×10^{-31}**
**PC ae C40:6**	-0.08 (-0.09, -0.07)	**4.94×10^{-33}**	**2.51×10^{-32}**	-0.08 (-0.1, -0.07)	**2.07×10^{-28}**	**9.36×10^{-28}**
PC ae C42:1	-0.02 (-0.04, 0)	0.08	0.11	-0.01 (-0.03, 0.01)	0.18	0.22
PC ae C42:2	0.01 (-0.01, 0.03)	0.24	0.27	0.01 (-0.01, 0.03)	0.33	0.38
PC ae C42:3	-0.01 (-0.02, 0)	0.17	0.20	0 (-0.02, 0.01)	0.68	0.72
PC ae C42:4	0 (-0.01, 0.01)	0.82	0.83	0.02 (0.01, 0.04)	4.72×10^{-3}	**6.77×10^{-3}**
PC ae C42:5	-0.02 (-0.03, -0.01)	1.21×10^{-3}	**1.91×10^{-3}**	-0.02 (-0.03, 0)	0.05	0.06
**PC ae C44:3**	-0.07 (-0.08, -0.05)	**8.35×10^{-13}**	**2.31×10^{-12}**	-0.06 (-0.09, -0.04)	**1.19×10^{-8}**	**2.74×10^{-8}**
**PC ae C44:4**	0.02 (0.01, 0.03)	**1.36×10^{-3}**	**2.13×10^{-3}**	0.04 (0.02, 0.05)	**3.05×10^{-6}**	**5.73×10^{-6}**
**PC ae C44:5**	0.08 (0.07, 0.09)	**9.51×10^{-48}**	**6.45×10^{-47}**	0.09 (0.07, 0.1)	**8.62×10^{-34}**	**4.57×10^{-33}**
**PC ae C44:6**	0.05 (0.04, 0.06)	**9.75×10^{-17}**	**3.50×10^{-16}**	0.06 (0.05, 0.07)	**6.77×10^{-16}**	**2.02×10^{-15}**
**LysoPC a C16:0**	-0.15 (-0.17, -0.14)	**5.65×10^{-89}**	**1.72×10^{-87}**	-0.15 (-0.17, -0.13)	**4.19×10^{-67}**	**8.52×10^{-66}**
**LysoPC a C16:1**	-0.1 (-0.11, -0.09)	**2.52×10^{-55}**	**2.79×10^{-54}**	-0.1 (-0.12, -0.09)	**1.38×10^{-38}**	**8.41×10^{-38}**
**LysoPC a C17:0**	-0.11 (-0.13, -0.1)	**9.31×10^{-57}**	**1.14×10^{-55}**	-0.1 (-0.12, -0.09)	**1.58×10^{-32}**	**8.02×10^{-32}**
**LysoPC a C18:0**	-0.12 (-0.14, -0.11)	**1.23×10^{-49}**	**9.86×10^{-49}**	-0.14 (-0.16, -0.12)	**3.95×10^{-56}**	**5.35×10^{-55}**
**LysoPC a C18:1**	-0.08 (-0.09, -0.06)	**2.75×10^{-26}**	**1.24×10^{-25}**	-0.09 (-0.11, -0.07)	**1.43×10^{-26}**	**6.22×10^{-26}**
**LysoPC a C18:2**	-0.06 (-0.08, -0.04)	**4.39×10^{-14}**	**1.34×10^{-13}**	-0.08 (-0.1, -0.07)	**6.18×10^{-23}**	**2.36×10^{-22}**
**LysoPC a C20:3**	-0.03 (-0.04, -0.01)	8.44×10^{-4}	**1.37×10^{-3}**	-0.05 (-0.07, -0.03)	**5.79×10^{-8}**	**1.24×10^{-7}**
**LysoPC a C20:4**	-0.01 (-0.03, 0)	0.04	0.05	-0.03 (-0.05, -0.02)	**4.82×10^{-5}**	**7.94×10^{-5}**
**SM (OH) C14:1**	-0.09 (-0.1, -0.08)	**7.23×10^{-50}**	**6.78×10^{-49}**	-0.1 (-0.11, -0.08)	**3.46×10^{-46}**	**3.01×10^{-45}**
**SM (OH) C16:1**	-0.08 (-0.09, -0.07)	**5.95×10^{-35}**	**3.30×10^{-34}**	-0.09 (-0.1, -0.07)	**3.07×10^{-37}**	**1.79×10^{-36}**
**SM (OH) C22:1**	-0.12 (-0.14, -0.11)	**2.75×10^{-79}**	**4.79×10^{-78}**	-0.14 (-0.15, -0.12)	**1.36×10^{-73}**	**3.33×10^{-72}**
**SM (OH) C22:2**	-0.09 (-0.1, -0.08)	**1.29×10^{-49}**	**9.86×10^{-49}**	-0.09 (-0.11, -0.08)	**4.56×10^{-41}**	**3.27×10^{-40}**
**SM (OH) C24:1**	-0.15 (-0.16, -0.14)	**1.82×10^{-113}**	**7.41×10^{-112}**	-0.16 (-0.17, -0.15)	**1.35×10^{-106}**	**8.24×10^{-105}**
**SM C16:0**	-0.07 (-0.09, -0.06)	**1.81×10^{-21}**	**7.36×10^{-21}**	-0.08 (-0.1, -0.07)	**2.54×10^{-23}**	**9.99×10^{-23}**
**SM C16:1**	-0.09 (-0.11, -0.08)	**1.34×10^{-48}**	**9.59×10^{-48}**	-0.11 (-0.12, -0.09)	**3.76×10^{-53}**	**4.59×10^{-52}**
**SM C18:0**	-0.06 (-0.07, -0.04)	**4.06×10^{-16}**	**1.41×10^{-15}**	-0.08 (-0.09, -0.06)	**1.71×10^{-20}**	**6.33×10^{-20}**
**SM C18:1**	-0.07 (-0.09, -0.06)	**2.15×10^{-30}**	**1.05×10^{-29}**	-0.09 (-0.1, -0.08)	**1.00×10^{-38}**	**6.45×10^{-38}**
**SM C20:2**	-0.16 (-0.18, -0.15)	**6.90×10^{-135}**	**4.21×10^{-133}**	-0.16 (-0.18, -0.15)	**8.70×10^{-104}**	**3.54×10^{-102}**
**SM C24:0**	-0.12 (-0.14, -0.11)	**3.75×10^{-68}**	**5.71×10^{-67}**	-0.15 (-0.16, -0.13)	**5.40×10^{-76}**	**1.65×10^{-74}**
**SM C24:1**	-0.12 (-0.14, -0.11)	**5.49×10^{-82}**	**1.12×10^{-80}**	-0.13 (-0.15, -0.12)	**3.80×10^{-65}**	**6.62×10^{-64}**
**SM C26:1**	-0.12 (-0.13, -0.1)	**3.18×10^{-67}**	**4.32×10^{-66}**	-0.13 (-0.15, -0.12)	**5.74×10^{-51}**	**5.84×10^{-50}**
**Sum of Hexoses**	-0.07 (-0.09, -0.05)	**1.10×10^{-14}**	**3.52×10^{-14}**	-0.07 (-0.09, -0.05)	**2.56×10^{-15}**	**7.45×10^{-15}**
**Arginine**	-0.1 (-0.12, -0.09)	**3.23×10^{-30}**	**1.52×10^{-29}**	-0.12 (-0.14, -0.09)	**5.10×10^{-29}**	**2.39×10^{-28}**
Glutamine	0 (-0.02, 0.02)	0.93	0.93	-0.01 (-0.04, 0.01)	0.29	0.33
Glycine	0.02 (0, 0.03)	0.02	**0.02**	0 (-0.01, 0.02)	0.70	0.73
**Histidine**	0.13 (0.12, 0.15)	**7.86×10^{-51}**	**7.99×10^{-50}**	0.15 (0.13, 0.17)	**1.21×10^{-48}**	**1.13×10^{-47}**
**Methionine**	-0.07 (-0.08, -0.05)	**2.07×10^{-12}**	**5.38×10^{-12}**	-0.09 (-0.11, -0.07)	**1.93×10^{-19}**	**6.74×10^{-19}**
**Ornithine**	0.14 (0.13, 0.16)	**4.58×10^{-82}**	**1.12×10^{-80}**	0.14 (0.12, 0.16)	**2.72×10^{-56}**	**4.15×10^{-55}**
**Phenylalanine**	-0.07 (-0.09, -0.05)	**6.55×10^{-13}**	**1.86×10^{-12}**	-0.09 (-0.11, -0.07)	**2.19×10^{-17}**	**7.02×10^{-17}**

**Proline**	-0.08 (-0.09, -0.07)	**1.28 x 10^{-34}**	**6.79 x 10^{-34}**	-0.1 (-0.11, -0.08)	**5.06 x 10^{-44}**	**3.86 x 10^{-43}**
**Serine**	0.04 (0.02, 0.06)	**2.96 x 10^{-4}**	**5.08 x 10^{-4}**	0.03 (0.01, 0.05)	4.99 x 10^{-3}	**7.08 x 10^{-3}**
**Threonine**	0.05 (0.03, 0.07)	**1.90 x 10^{-7}**	**3.79 x 10^{-7}**	0.06 (0.03, 0.08)	**5.90 x 10^{-7}**	**1.18 x 10^{-6}**
**Tryptophan**	-0.04 (-0.06, -0.02)	**4.76 x 10^{-5}**	**8.66 x 10^{-5}**	-0.09 (-0.11, -0.07)	**3.01 x 10^{-19}**	**1.02 x 10^{-18}**
**Tyrosine**	0.11 (0.09, 0.13)	**1.58 x 10^{-36}**	**9.19 x 10^{-36}**	0.07 (0.06, 0.09)	**2.93 x 10^{-16}**	**8.95 x 10^{-16}**
**Valine**	-0.04 (-0.05, -0.02)	**1.45 x 10^{-5}**	**2.73 x 10^{-5}**	-0.06 (-0.08, -0.04)	**3.23 x 10^{-8}**	**7.16 x 10^{-8}**
**xLeucine**	-0.2 (-0.21, -0.18)	**1.46 x 10^{-219}**	**1.78 x 10^{-217}**	-0.22 (-0.23, -0.21)	**8.63 x 10^{-228}**	**1.05 x 10^{-225}**

Table S3. Replication results of the changes in metabolite concentration in CARLA study.

The table shows the replication results in CARLA study based on the 72 and 81 significant metabolites identified in discovery (KORA) study in women and men respectively. Metabolites that were not available for replication due to limit of detection (LOD) were indicated. Beta estimates (ß) and confidence interval (CI) of changes in metabolite concentration every year were calculated using multivariate generalised estimation equation models. The model was adjusted for chronological age at baseline, body mass index, physical activity, smoking status, alcohol intake and systolic blood pressure. Significant P values after Bonferroni correction (cut-offs: P value < $\frac{0.05}{53}$ = 9.5 x 10^{-4} in women and P value < $\frac{0.05}{58}$ = 8.7 x 10^{-4} in men) and their false discovery rate-adjusted P values (pFDR) at 5% level were highlighted in bold. xLeucine, sum of leucine and isoleucine.

	**Women in CARLA (*N* = 195)**			**Men in CARLA (*N* = 191)**		
**Metabolites**	**ß (95% CI)**	**P value**	**pFDR**	**ß (95% CI)**	**P value**	**pFDR**
C0	0.04 (0.005, 0.068)	0.02	0.09	0.01 (-0.012, 0.042)	0.29	0.46
C2	0.05 (0.013, 0.094)	0.01	0.05	0.03 (-0.011, 0.061)	0.17	0.36
C3	0.02 (-0.01, 0.056)	0.17	0.33	0 (-0.027, 0.033)	0.84	0.88
C4	Not in dataset. LOD			Not in dataset. LOD		
C5	0.02 (-0.018, 0.052)	0.35	0.50	0.02 (-0.015, 0.062)	0.23	0.41
C10:1	0.01 (-0.02, 0.046)	0.44	0.54	-0.01 (-0.042, 0.025)	0.62	0.76
C12	0.04 (0.006, 0.076)	0.02	0.09	-0.01 (-0.041, 0.028)	0.73	0.80
C14	Not in dataset. LOD			Not in dataset. LOD		
C14:1	Not in dataset. LOD			Not in dataset. LOD		
C14:2	0.04 (-0.004, 0.081)	0.08	0.21	0.03 (-0.011, 0.066)	0.16	0.35
C16:1	Not in dataset. LOD			Not in dataset. LOD		
**C18**	0.03 (0.019, 0.04)	**6.05 x 10^{-8}**	**1.21 x 10^{-6}**	0.02 (0.009, 0.029)	**2.81 x 10^{-4}**	**2.81 x 10^{-3}**
**C18:1**	0.12 (0.083, 0.159)	**5.20 x 10^{-10}**	**1.51 x 10^{-8}**	0.11 (0.073, 0.144)	**1.67 x 10^{-9}**	**4.85 x 10^{-8}**
PC aa C30:0	0.02 (-0.017, 0.057)	0.29	0.44	0,44	0.59	0.29
PC aa C32:0	0.01 (-0.017, 0.044)	0.39	0.52	0,09	0.28	0.39
**PC aa C32:1**	0.02 (-0.007, 0.052)	0.13	0.28	-0.04 (-0.071, -0.015)	2.57 x 10^{-3}	**0.02**
PC aa C34:1	Not in dataset. LOD			Not in dataset. LOD		
PC aa C36:0	Not in dataset. LOD			Not in dataset. LOD		
PC aa C36:2	Not in dataset. LOD			Not in dataset. LOD		
**PC aa C36:3**	0.01 (-0.022, 0.036)	0.65	0.75	-0.05 (-0.078, -0.017)	2.31 x 10^{-3}	**0.02**
PC aa C38:0	0 (-0.028, 0.028)	1.00	1.00	-0.04 (-0.067, -0.007)	0.01	0.09
PC aa C38:3	0.02 (-0.006, 0.055)	0.11	0.27	-0.04 (-0.069, -0.007)	0.02	0.09
PC aa C38:4	0.03 (-0.006, 0.06)	0.12	0.28	-0.03 (-0.064, -0.004)	0.03	0.12
PC aa C40:5	Not in dataset. LOD			Not in dataset. LOD		
PC aa C40:6	Not in dataset. LOD			Not in dataset. LOD		
PC aa C42:0	0 (-0.026, 0.028)	0.95	0.96	-0.01 (-0.042, 0.013)	0.31	0.47
PC aa C42:1	0.01 (-0.021, 0.034)	0.65	0.75	-0.01 (-0.04, 0.019)	0.48	0.62
PC aa C42:4	0.02 (-0.017, 0.058)	0.29	0.44	-0.01 (-0.047, 0.021)	0.44	0.59
PC aa C42:6	Not in dataset. LOD			Not in dataset. LOD		
PC ae C32:1	0.02 (-0.007, 0.056)	0.13	0.28	-0.02 (-0.051, 0.016)	0.30	0.47
PC ae C32:2	0 (-0.026, 0.033)	0.84	0.89	-0.02 (-0.051, 0.006)	0.12	0.30
PC ae C34:0	Not in dataset. LOD			Not in dataset. LOD		
PC ae C34:1	Not in dataset. LOD			Not in dataset. LOD		
PC ae C34:2	Not in dataset. LOD			Not in dataset. LOD		
PC ae C34:3	0.01 (-0.017, 0.044)	0.40	0.52	-0.03 (-0.055, 0.005)	0.10	0.28
PC ae C36:1	Not in dataset. LOD			Not in dataset. LOD		
PC ae C36:3	Not in dataset. LOD			Not in dataset. LOD		
PC ae C36:5	0.02 (-0.007, 0.052)	0.13	0.28	-0.02 (-0.049, 0.013)	0.25	0.43
PC ae C38:1	Not in dataset. LOD			Not in dataset. LOD		
PC ae C38:3	Not in dataset. LOD			Not in dataset. LOD		
PC ae C38:4	Not in dataset. LOD			Not in dataset. LOD		
PC ae C38:6	Not in dataset. LOD			Not in dataset. LOD		
PC ae C40:2	Not in dataset. LOD			Not in dataset. LOD		
PC ae C40:3	0 (-0.029, 0.039)	0.78	0.84	-0.04 (-0.066, -0.005)	0.02	0.10
PC ae C40:4	0.02 (-0.013, 0.05)	0.24	0.41	-0.03 (-0.059, 0.005)	0.10	0.28

**PC ae C40:5**	0 (-0.035, 0.031)	0.89	0.93	-0.06 (-0.091, -0.025)	**5.36 x 10^{-4}**	**6.22 x 10^{-3}**
PC ae C40:6	-0.01 (-0.038, 0.025)	0.67	0.75	-0.04 (-0.076, -0.012)	7.53 x 10^{-3}	0.05
PC ae C44:3	-0.01 (-0.043, 0.021)	0.51	0.62	0 (-0.032, 0.039)	0.85	0.88
PC ae C44:4	0.04 (0.005, 0.066)	0.02	0.09	-0.02 (-0.046, 0.01)	0.22	0.41
PC ae C44:5	0.03 (0, 0.051)	0.05	0.15	0 (-0.026, 0.025)	0.97	0.97
PC ae C44:6	0.02 (-0.009, 0.044)	0.20	0.37	-0.01 (-0.037, 0.016)	0.45	0.59
**LysoPC a C16:0**	0.06 (0.034, 0.09)	**1.59 x 10^{-5}**	**2.31 x 10^{-4}**	0.02 (-0.004, 0.048)	0.10	0.28
LysoPC a C16:1	0.03 (0, 0.06)	0.05	0.15	0 (-0.031, 0.028)	0.92	0.94
LysoPC a C17:0	0.03 (0.004, 0.062)	0.03	0.09	0.02 (-0.01, 0.046)	0.20	0.39
**LysoPC a C18:0**	0.08 (0.053, 0.116)	**1.43 x 10^{-7}**	**2.77 x 10^{-6}**	0.02 (-0.006, 0.049)	0.12	0.30
LysoPC a C18:1	0.01 (-0.02, 0.048)	0.42	0.53	-0.01 (-0.038, 0.025)	0.67	0.78
LysoPC a C18:2	-0.02 (-0.049, 0.018)	0.36	0.50	-0.04 (-0.071, -0.006)	0.02	0.10
LysoPC a C20:3	0.01 (-0.028, 0.043)	0.69	0.75	-0.03 (-0.065, -0.001)	0.05	0.18
LysoPC a C20:4	0.03 (-0.009, 0.061)	0.15	0.30	-0.01 (-0.038, 0.027)	0.75	0.80
SM (OH) C14:1	0.03 (0.001, 0.055)	0.04	0.13	-0.01 (-0.027, 0.011)	0.41	0.58
SM (OH) C16:1	0.02 (-0.01, 0.043)	0.21	0.37	-0.01 (-0.027, 0.017)	0.64	0.76
SM (OH) C22:1	0.02 (-0.009, 0.042)	0.21	0.37	-0.02 (-0.038, 0.004)	0.12	0.30
SM (OH) C22:2	0.01 (-0.011, 0.039)	0.27	0.44	-0.01 (-0.032, 0.009)	0.28	0.46
SM (OH) C24:1	Not in dataset. LOD			Not in dataset. LOD		
SM C16:0	0.03 (0.004, 0.058)	0.02	0.09	-0.01 (-0.027, 0.017)	0.64	0.76
SM C16:1	0.02 (-0.007, 0.05)	0.15	0.30	-0.01 (-0.034, 0.012)	0.35	0.51
SM C18:0	0.02 (-0.004, 0.053)	0.10	0.25	0 (-0.03, 0.021)	0.74	0.80
SM C18:1	0.01 (-0.022, 0.034)	0.67	0.75	-0.02 (-0.042, 0.007)	0.17	0.35
SM C20:2	Not in dataset. LOD			Not in dataset. LOD		
SM C24:0	0.01 (-0.014, 0.038)	0.36	0.50	-0.02 (-0.044, -0.001)	0.04	0.15
SM C24:1	0.01 (-0.015, 0.04)	0.38	0.51	-0.02 (-0.044, 0.007)	0.16	0.35
SM C26:1	Not in dataset. LOD			Not in dataset. LOD		
Sum of Hexoses	Not in dataset. LOD			Not in dataset. LOD		
**Arginine**	-0.06 (-0.1, -0.025)	1.05 x 10^{-3}	**1.01 x 10^{-2}**	-0.07 (-0.101, -0.034)	**7.03 x 10^{-5}**	**1.36 x 10^{-3}**
Histidine	0.05 (0.012, 0.08)	8.28 x 10^{-3}	0.05	0.01 (-0.026, 0.048)	0.56	0.71
Methionine	Not in dataset. LOD			Not in dataset. LOD		
**Ornithine**	0.24 (0.207, 0.272)	**6.86 x 10^{-48}**	**3.98 x 10^{-46}**	0.22 (0.186, 0.25)	**2.78 x 10^{-41}**	**1.61 x 10^{-39}**
**Phenylalanine**	0.07 (0.032, 0.106)	**2.84 x 10^{-4}**	**3.29 x 10^{-3}**	0.07 (0.032, 0.099)	**1.28 x 10^{-4}**	**1.86 x 10^{-3}**
Proline	0.04 (0.01, 0.071)	9.67 x 10^{-3}	0.05	0.01 (-0.015, 0.044)	0.33	0.49
**Serine**	0.01 (0.007, 0.022)	1.14 x 10^{-3}	**0.01**	0.02 (0.01, 0.02)	**3.85 x 10^{-7}**	**7.71 x 10^{-6}**
Threonine	0.04 (0.002, 0.07)	0.04	0.13	0.03 (-0.001, 0.065)	0.06	0.20
Tryptophan	0.02 (-0.017, 0.053)	0.31	0.46	-0.01 (-0.037, 0.025)	0.73	0.80
**Tyrosine**	0.05 (0.013, 0.084)	6.80 x 10^{-3}	**4.93 x 10^{-2}**	0.03 (-0.001, 0.064)	0.06	0.20
**Valine**	0.04 (0.016, 0.067)	1.22 x 10^{-3}	**0.01**	0.02 (-0.008, 0.04)	0.19	0.38
xLeucine	0.02 (-0.018, 0.062)	0.29	0.44	0.02 (-0.017, 0.062)	0.27	0.45

Table S4. The list of metabolite concentration of 122 metabolites analyzed in KORA S4 and F4.

The list shows the mean (in µM), standard deviation and median values (in µM of all 122 metabolites measured in both KORA S4 and F4. The metabolite concentration values shown in KORA F4 were after inter-kit normalisation by multiplying the concentration values to the NF of their corresponding metabolites. SD, standard deviation. xLeucine, sum of leucine and isoleucine.

	Women in KORA (n = 317)						Men in KORA (n = 273)					
	KORA S4			KORA F4			KORA S4			KORA F4		
Marker	Mean	SD	Median	Mean	SD	Median	Mean	SD	Median	Mean	SD	Median
C0	38.3	8.47	37.9	34.63	6.45	33.89	42.59	7.94	42.3	36.55	7.19	35.81
C2	8.25	2.71	7.7	9.32	2.85	8.75	8.35	3.12	7.79	9.19	3.18	8.56
C3	0.43	0.14	0.41	0.38	0.1	0.36	0.5	0.16	0.48	0.42	0.16	0.4
C4	0.22	0.11	0.19	0.24	0.11	0.21	0.25	0.17	0.21	0.25	0.15	0.21
C6 (C4:1-DC)	0.08	0.03	0.08	0.09	0.03	0.08	0.09	0.03	0.08	0.09	0.03	0.08
C5	0.15	0.09	0.14	0.12	0.04	0.11	0.17	0.04	0.17	0.14	0.05	0.13
C8	0.25	0.1	0.23	0.25	0.09	0.23	0.26	0.09	0.24	0.26	0.09	0.24
C10	0.36	0.18	0.32	0.37	0.14	0.34	0.38	0.16	0.35	0.38	0.15	0.34
C10:1	0.17	0.06	0.16	0.16	0.05	0.15	0.18	0.06	0.17	0.16	0.05	0.15
C10:2	0.04	0.01	0.04	0.04	0.01	0.04	0.04	0.01	0.04	0.04	0.01	0.04
C12	0.15	0.06	0.14	0.16	0.06	0.15	0.16	0.06	0.16	0.18	0.07	0.16
C14	0.05	0.02	0.05	0.05	0.01	0.05	0.06	0.01	0.06	0.05	0.01	0.05
C14:1	0.14	0.05	0.13	0.17	0.04	0.16	0.15	0.05	0.14	0.17	0.05	0.16
C14:2	0.04	0.02	0.03	0.03	0.01	0.03	0.04	0.02	0.04	0.04	0.02	0.03
C16	0.13	0.03	0.12	0.13	0.03	0.12	0.14	0.03	0.14	0.14	0.03	0.14
C16:1	0.04	0.01	0.04	0.04	0.01	0.04	0.04	0.01	0.04	0.04	0.01	0.04
C18	0.05	0.01	0.05	0.05	0.01	0.05	0.06	0.01	0.06	0.06	0.01	0.06
C18:1	0.13	0.04	0.13	0.13	0.03	0.12	0.15	0.04	0.14	0.13	0.04	0.13
C18:2	0.05	0.01	0.04	0.05	0.01	0.05	0.05	0.02	0.05	0.05	0.02	0.05
PC aa C28:1	3.98	0.84	3.85	4.01	0.93	3.85	3.39	0.71	3.38	3.33	0.74	3.31
PC aa C30:0	6.3	1.63	6.07	5.47	1.56	5.26	5.73	1.68	5.43	4.86	1.47	4.63
PC aa C32:0	15.04	2.58	14.7	15.99	2.93	15.88	14.88	2.9	14.6	15.66	3.26	15.43
PC aa C32:1	21.21	9.64	19	23.71	9.42	21.79	19.91	10.72	17.3	22.1	12.36	19.37
PC aa C32:2	4.94	1.83	4.8	4.8	1.92	4.62	3.99	1.37	3.81	3.96	1.76	3.81
PC aa C32:3	0.62	0.14	0.61	0.59	0.13	0.58	0.49	0.12	0.48	0.47	0.12	0.47
PC aa C34:1	225.99	42.45	225	241.43	49.59	237.53	220.46	49.24	216	234.47	52.45	232.03
PC aa C34:2	373.13	45.58	371	367.07	54.77	363.56	360.75	51.96	355	353.33	60.2	348.59
PC aa C34:3	20.05	5.04	19.6	19.61	4.67	19.1	17.17	4.7	16.7	16.7	4.81	15.86
PC aa C34:4	2.44	0.8	2.37	2.53	0.83	2.42	2.04	0.69	1.94	2.06	0.73	1.96
PC aa C36:0	3.1	0.76	3.01	2.51	0.81	2.38	3.01	0.77	2.89	2.42	0.87	2.3
PC aa C36:1	55.35	13.11	54.8	55.77	12.49	54.48	52.37	14.33	49.9	51.35	13.73	48.91
PC aa C36:2	261.92	39.88	262	225.99	36.59	225.26	248.99	43.34	246	208.86	38.37	206.41
PC aa C36:3	158.51	29.21	154	147.27	25.32	147.13	145.86	27.93	144	134.94	27.2	132.89
PC aa C36:4	215.25	42.88	211	215.23	43.48	210.91	203.48	43.36	200	202.07	46.7	198.02
PC aa C36:5	31.83	14.14	29.3	30.87	13.86	27.07	31.3	16.21	27.4	29.24	15.97	25.19
PC aa C36:6	1.26	0.44	1.23	1.22	0.46	1.12	1.06	0.42	0.97	1.01	0.41	0.94
PC aa C38:0	3.51	0.88	3.39	3.32	0.99	3.21	3.33	0.91	3.15	3.11	0.92	2.96
PC aa C38:3	59.54	13.54	57.5	55.91	12.03	55.22	53.02	12.35	52.1	48.1	11.53	47.04
PC aa C38:4	121.81	27.98	120	120.27	27.8	117.99	112.03	27.06	110	107.12	27.95	104.44
PC aa C38:5	65.99	14.26	66	64.7	14.44	63.29	61.87	15.97	60.7	58.7	15.57	56.53
PC aa C38:6	94.19	26.13	91.3	92.13	27.55	87.87	88.48	26.53	83.1	86.18	24.91	84.55
PC aa C40:2	0.38	0.09	0.36	0.37	0.11	0.34	0.37	0.11	0.35	0.37	0.16	0.34
PC aa C40:3	0.7	0.15	0.68	0.69	0.17	0.65	0.67	0.16	0.65	0.67	0.22	0.63
PC aa C40:4	4.15	1.09	4	4.07	1.02	4.02	4.03	1.26	3.88	3.84	1.17	3.61
PC aa C40:5	12.9	3.29	12.8	11.7	3.05	11.42	12.52	4.01	11.9	10.69	2.96	10.2
PC aa C40:6	33.02	9.6	31.7	31.38	10.62	29.66	31.3	10.3	29.7	28.26	8.81	27.07
PC aa C42:0	0.59	0.15	0.56	0.66	0.2	0.62	0.56	0.17	0.52	0.63	0.23	0.59
PC aa C42:1	0.28	0.07	0.28	0.31	0.09	0.29	0.27	0.07	0.26	0.29	0.09	0.28
PC aa C42:2	0.21	0.05	0.2	0.22	0.06	0.21	0.21	0.06	0.2	0.22	0.07	0.21
PC aa C42:4	0.21	0.04	0.21	0.23	0.05	0.22	0.21	0.04	0.2	0.23	0.06	0.22
PC aa C42:5	0.45	0.12	0.43	0.44	0.13	0.41	0.42	0.15	0.39	0.43	0.17	0.4
PC aa C42:6	0.63	0.15	0.62	0.58	0.13	0.55	0.59	0.16	0.56	0.55	0.14	0.52
PC ae C30:0	0.5	0.13	0.49	0.48	0.15	0.46	0.43	0.12	0.41	0.44	0.16	0.41
PC ae C32:1	2.99	0.58	2.92	3.16	0.66	3.15	2.72	0.5	2.69	2.96	0.64	2.91
PC ae C32:2	0.79	0.16	0.77	0.87	0.2	0.86	0.66	0.13	0.65	0.75	0.18	0.73
PC ae C34:0	1.85	0.43	1.83	1.96	0.49	1.88	1.63	0.38	1.58	1.74	0.42	1.71
PC ae C34:1	11.46	2.09	11.3	11.77	2.37	11.75	9.91	1.84	9.81	10.44	2.22	10.36
PC ae C34:2	13.36	2.76	13.3	13.67	3.13	13.42	11.42	2.44	11.4	12.19	3	11.9
PC ae C34:3	8.47	2.19	8.17	9.21	2.48	9.06	7.41	1.83	7.1	8.3	2.35	7.88

PC ae C36:1	9.94	2	9.8	9.07	2.06	8.97	8.37	1.77	8.13	7.73	1.81	7.61
PC ae C36:2	17.25	3.67	17.2	16.85	3.7	16.55	14.3	3.15	14.1	14.29	3.47	13.96
PC ae C36:3	8.62	1.77	8.5	8.93	1.91	8.81	7.62	1.55	7.57	8.15	1.85	7.96
PC ae C36:4	20.36	4.51	19.7	20.3	4.77	19.55	19.89	4.48	19.3	20.1	4.82	19.44
PC ae C36:5	13.3	3.23	13	14.34	3.69	13.66	13.09	3.09	12.9	13.82	3.44	13.5
PC ae C38:0	2.46	0.66	2.43	2.37	0.75	2.28	2.16	0.67	2.05	2.07	0.7	1.9
PC ae C38:1	0.66	0.27	0.62	0.81	0.35	0.76	0.6	0.24	0.59	0.8	0.48	0.71
PC ae C38:2	2.3	0.47	2.27	2.23	0.49	2.2	2.03	0.42	1.96	2	0.54	1.96
PC ae C38:3	4.76	0.94	4.69	4.51	0.96	4.45	3.92	0.78	3.88	3.76	0.79	3.71
PC ae C38:4	16.46	3.08	16.2	15.43	3.05	15.01	14.9	2.69	14.6	14.18	2.88	13.9
PC ae C38:5	19.53	3.72	19.2	19.4	4.03	18.8	19.32	3.63	19.1	19.05	4.14	18.7
PC ae C38:6	9.15	2.09	8.89	8.89	2.26	8.65	8.63	2.06	8.33	8.24	2.08	8.08
PC ae C40:1	1.69	0.37	1.66	1.75	0.38	1.71	1.61	0.38	1.55	1.65	0.41	1.59
PC ae C40:2	2.31	0.48	2.27	2.23	0.53	2.18	2.07	0.47	2.01	1.97	0.44	1.93
PC ae C40:3	1.31	0.22	1.3	1.18	0.25	1.16	1.11	0.2	1.08	1.03	0.24	0.99
PC ae C40:4	2.87	0.51	2.84	2.65	0.52	2.59	2.62	0.47	2.59	2.49	0.61	2.4
PC ae C40:5	3.89	0.7	3.88	3.39	0.68	3.35	3.65	0.63	3.58	3.2	0.69	3.15
PC ae C40:6	5.91	1.38	5.68	5.12	1.38	4.94	5.34	1.25	5.22	4.64	1.2	4.49
PC ae C42:1	0.4	0.09	0.39	0.39	0.09	0.37	0.38	0.09	0.37	0.37	0.11	0.35
PC ae C42:2	0.68	0.14	0.67	0.68	0.15	0.68	0.63	0.13	0.61	0.63	0.14	0.62
PC ae C42:3	0.91	0.18	0.91	0.9	0.21	0.87	0.85	0.19	0.83	0.85	0.21	0.83
PC ae C42:4	1.01	0.22	0.99	1.01	0.24	0.98	0.94	0.23	0.92	0.98	0.34	0.93
PC ae C42:5	2.3	0.46	2.24	2.22	0.49	2.15	2.2	0.47	2.1	2.16	0.64	2.07
PC ae C44:3	0.13	0.04	0.13	0.11	0.03	0.11	0.13	0.04	0.12	0.11	0.03	0.11
PC ae C44:4	0.4	0.1	0.4	0.41	0.11	0.4	0.38	0.1	0.37	0.4	0.14	0.38
PC ae C44:5	1.79	0.46	1.72	2.08	0.57	1.99	1.77	0.48	1.69	2.12	0.78	1.99
PC ae C44:6	1.31	0.33	1.25	1.44	0.39	1.37	1.27	0.36	1.19	1.44	0.5	1.36
LysoPC a C16:0	120.17	25.15	116	93.02	18.15	91.84	131.42	24.7	129	101.92	20.61	98.95
LysoPC a C16:1	3.64	1.01	3.49	2.91	0.82	2.84	3.87	1.44	3.65	3.05	1.2	2.81
LysoPC a C17:0	2.29	0.69	2.18	1.78	0.48	1.73	2.16	0.62	2.11	1.73	0.51	1.66
LysoPC a C18:0	32.58	8.34	31.6	25.63	6.03	25.22	34.38	7.45	33.5	26.44	6.11	26.05
LysoPC a C18:1	20.75	5.5	20	17.66	4.31	17.04	24.29	6.66	23.6	20.07	5.79	19.25
LysoPC a C18:2	26.72	8.07	25.2	23.12	5.89	22.68	33.13	9.39	31.5	27.4	8.11	26.29
LysoPC a C20:3	2.17	0.6	2.08	2.06	0.54	2.01	2.46	0.66	2.45	2.19	0.61	2.18
LysoPC a C20:4	5.81	1.65	5.63	5.68	1.63	5.54	6.93	1.97	6.62	6.42	1.81	6.36
SM (OH) C14:1	10.71	2.41	10.5	8.95	2.11	8.75	8.73	2.08	8.65	7.29	1.81	7.12
SM (OH) C16:1	5.78	1.25	5.69	4.98	1.13	4.94	4.74	1.14	4.71	4.03	0.97	3.91
SM (OH) C22:1	22.43	4.27	22.2	17.92	3.67	17.86	18.91	3.85	18.9	14.74	3.22	14.46
SM (OH) C22:2	19.08	3.6	19	16.19	3.42	15.9	14.68	3.12	14.4	12.4	2.76	12.06
SM (OH) C24:1	2.14	0.49	2.11	1.58	0.37	1.54	1.92	0.45	1.88	1.38	0.33	1.33
SM C16:0	156.3	23.5	156	142.81	21.72	141.86	145.98	23.52	142	131.83	22.05	130.55
SM C16:1	25.85	4.4	25.8	22.54	3.58	22.33	21.67	3.61	21.3	18.52	3.47	18.57
SM C18:0	35.07	6.76	34.4	31.95	5.97	32.08	30.46	6.42	30.4	27.12	5.49	26.91
SM C18:1	18.45	4	17.95	16.11	3.08	15.84	14.64	3.26	14.5	12.52	2.79	12.43
SM C20:2	0.75	0.24	0.72	0.46	0.14	0.46	0.57	0.18	0.54	0.35	0.1	0.34
SM C24:0	30.66	5.41	30.8	25.25	4.89	25.13	30.07	5.63	29.8	23.72	4.89	23.37
SM C24:1	78.2	14.25	77.7	64.8	12.68	64.09	75.29	14.78	73.4	60.95	11.81	60.89
SM C26:1	0.65	0.18	0.64	0.5	0.14	0.48	0.65	0.21	0.63	0.48	0.14	0.46
Sum of Hexoses	5002.81	601.04	4929	4704.12	604.46	4657.44	5148.81	495.12	5097	4861.37	628.25	4820.87
Arginine	124.52	26.43	122	104.43	16.99	102.92	125.54	25.18	122	104	18.3	103.78
Glutamine	573.7	116.26	562	565.31	75.34	560.86	590.83	116.17	572	576.81	90.63	571.27
Glycine	284.92	87.62	269	291.98	78.74	273.39	247.58	57.22	238	246.12	51.07	240.44
Histidine	82.48	14.85	81.2	96.75	12.69	95.29	82.51	13.83	81.2	98.79	14.89	98
Methionine	22.2	4.36	21.8	19.97	3.28	19.8	25.01	5.16	24.5	21.71	4.09	21.62
Ornithine	56.87	15	54.2	73.86	14.61	73.24	58.94	13.33	57.5	77	17.76	76.58
Phenylalanine	71.74	14.7	70.7	64.47	9.21	63.53	77.63	15.23	75.9	68.09	11.76	67.13
Proline	170.47	54.67	165	143.34	47.78	133.86	208.2	58.62	196	170.05	47.51	159.64
Serine	129.29	30.77	128	134.42	23.8	132.08	122.35	25.15	121	127.54	23.51	126.07
Threonine	119.59	29.76	118	127.61	27.58	124.93	122.21	31.35	117	132.04	28.2	131.03
Tryptophan	56.84	11.46	56.1	53.33	5.74	53.24	62.43	11.56	61.3	55.31	7.15	55.08
Tyrosine	66.48	17.29	65.2	78.4	15.24	77.6	72.59	17.04	69.1	81.7	17.69	79.76
Valine	204.48	41.72	200	192.31	31.46	192.01	235.25	47.51	230	216.03	38.14	212.51
Leucine	199.56	43.62	195.6	127	20.84	124.45	249.21	54.52	244.5	150.46	25.79	149.5
